# Advance directives and power of attorney for health care in the oldest-old – results of the AgeQualiDe study

**DOI:** 10.1186/s12877-017-0482-8

**Published:** 2017-04-13

**Authors:** Tobias Luck, Francisca S. Rodriguez, Birgitt Wiese, Carolin van der Leeden, Kathrin Heser, Horst Bickel, Jürgen in der Schmitten, Hans-Helmut Koenig, Siegfried Weyerer, Silke Mamone, Tina Mallon, Michael Wagner, Dagmar Weeg, Angela Fuchs, Christian Brettschneider, Jochen Werle, Martin Scherer, Wolfgang Maier, Steffi G. Riedel-Heller, for the AgeCoDe & AgeQualiDe study group, Wolfgang Maier, Martin Scherer, Steffi G. Riedel-Heller, Heinz-Harald Abholz, Christian Brettschneider, Cadja Bachmann, Horst Bickel, Wolfgang Blank, Sandra Eifflaender-Gorfer, Marion Eisele, Annette Ernst, Angela Fuchs, André Hajek, Kathrin Heser, Frank Jessen, Hanna Kaduszkiewicz, Teresa Kaufeler, Mirjam Köhler, Hans-Helmut König, Alexander Koppara, Diana Lubisch, Tobias Luck, Dagmar Lühmann, Melanie Luppa, Tina Mallon, Manfred Mayer, Edelgard Mösch, Michael Pentzek, Jana Prokein, Alfredo Ramirez, Susanne Roehr, Anna Schumacher, Janine Stein, Susanne Steinmann, Franziska Tebarth, Hendrik van den Bussche, Carolin van der Leeden, Michael Wagner, Klaus Weckbecker, Dagmar Weeg, Jochen Werle, Siegfried Weyerer, Birgitt Wiese, Steffen Wolfsgruber, Thomas Zimmermann

**Affiliations:** 1 Institute of Social Medicine, Occupational Health and Public Health (ISAP), University of Leipzig, Philipp-Rosenthal-Str. 55, 04103 Leipzig, Germany; 2 LIFE – Leipzig Research Center for Civilization Diseases, Universität Leipzig, Leipzig, Germany; 3 Edward R. Roybal Institute on Aging, University of Southern California, Los Angeles, USA; 4 Work Group Medical Statistics and IT-Infrastructure, Institute for General Practice, Hannover Medical School, Hannover, Germany; 5 Department of Primary Medical Care, Center for Psychosocial Medicine, University Medical Center Hamburg-Eppendorf, Hamburg, Germany; 6 Department of Psychiatry, University of Bonn, Bonn, Germany; 7 Department of Psychiatry, Klinikum rechts der Isar, Technical University of Munich, Munich, Germany; 8 Institute of General Practice, Medical Faculty, Heinrich-Heine-University Düsseldorf, Düsseldorf, Germany; 9 Department of Health Economics and Health Services Research, Hamburg Center for Health Economics, University Medical Center Hamburg-Eppendorf, Hamburg, Germany; 10 Central Institute of Mental Health, Medical Faculty Mannheim/Heidelberg University, Mannheim, Germany; 11 DZNE, German Center for Neurodegenerative Diseases, Bonn, Germany

**Keywords:** Oldest-old age, Prevalence, Frequency, Primary care, Power of attorney, Advance directives, Advance care planning

## Abstract

**Background:**

Completion of advance directives (ADs) and power of attorney (POA) documents may protect a person’s autonomy in future health care situations when the individual lacks decisional capacity. As such situations become naturally much more common in old age, we specifically aimed at providing information on (i) the frequency of ADs/POA in oldest-old individuals and (ii) factors associated with having completed ADs/POA.

**Methods:**

We analyzed data of oldest-old primary care patients (85+ years; including community-dwelling and institutionalized individuals) within the German AgeQualiDe study. Patients were initially recruited via their general practitioners (GPs). We calculated frequencies of ADs and POA for health care with 95% confidence intervals (CI) and used multivariable logistic regression analysis to evaluate the association between having ADs and POA and participants’ socio-demographic, cognitive, functional, and health-related characteristics.

**Results:**

Among 868 GP patients participating in AgeQualiDe (response = 90.9%), *n* = 161 had dementia and *n* = 3 were too exhausted/ill to answer the questions. Out of the remaining 704 (81.1%) dementia-free patients (mean age = 88.7 years; SD = 3.0), 69.0% (95%-CI = 65.6–72.4) stated to having ADs and 64.6% (95%-CI = 61.1–68.2) to having a POA for health care. Individual characteristics did not explain much of the variability of the presence/absence of ADs and POA (regression models: Nagelkerke’s R^2^ = 0.034/0.051). The most frequently stated reasons for not having ADs were that the older adults trust their relatives or physicians to make the right decisions for them when necessary (stated by 59.4% and 44.8% of those without ADs). Among the older adults with ADs, the majority had received assistance in its preparation (79.0%), most frequently from their children/grandchildren (38.3%). Children/grandchildren were also the most frequently stated group of designated persons (76.7%) for those with a POA for health care.

**Conclusions:**

Our findings suggest a high dissemination of ADs and POA for health care in the oldest-old in Germany. Some adults without ADs/POA perhaps would have completed advance care documents, if they had had received more information and support. When planning programs to offer advanced care planning to the oldest old, it might be helpful to respond to these specific needs, and also to be sensitive to attitudinal differences in this target group.

## Background

Technological-medical advances regarding terminal illness and end-of-life care have led to an increasing number of situations requiring serious decisions by patients, families and health care providers (e.g., regarding certain life-sustaining measures). In these situations, the patient itself, however, often has reduced or no decisional capacity (e.g., because of coma or cognitive deficits). Rising life expectancy – associated with an increased prevalence of cognitive deficits up to clinically manifest dementia [1] – may even lead to an increasing number of patients without decisional capacity. *Advance directives* (ADs), written documents that specify personal preferences for future medical care in the event that an individual loses decisional capacity, are a widely recognized tool that may help to protect a patient’s autonomy and intentions in such situations (e.g., better alignment between care as preferred and care as received, allowing/disallowing certain life-sustaining measures). The same holds true for a *power of attorney* (POA) for health care authorizing other persons to make medical decisions if an individual is temporarily or permanently unable to make own decisions (e.g. [2–5]). Both ADs and POA for health care are, moreover, part of the broader topic of *advance care planning* (ACP), “[…] a wider process including, for example, ongoing conversations between the competent adult and their family and health professionals about goals of future care.” (White et al. [5]; page 975). In general, offering ACP conversations is encouraged by promising findings demonstrating that the implementation of systematic ACP approaches can help to increase the prevalence as well as the quality of ADs [6, 7], and shows relevant clinical effects. In a randomized-controlled trial conducted by Detering et al. with *n* = 309 legally competent medical inpatients aged 80+ years, ACP improved end of life care, increased patient and family satisfaction and reduced stress, anxiety, and depression in relatives [8].

Yet, the completion of an ADs or the appointment of a POA seems to be rather uncommon. In Germany, for example, a critical review from 2012 that included 32 studies (conducted between 1996 and 2009) indicated increasing awareness of ADs but also several rather unsatisfying results such as low completion rates (e.g. 2.5–10% in the German general population) or fears about the purpose and possible abuse of ADs [9]. A study by Sommer et al. [10] showed low ADs rates in German nursing homes (11% of the residents had personal ADs and 1.4% ADs by proxy, data from 2007).

On September 1, 2009, a new law came into effect in Germany that, in brief, essentially confirmed the legality of written ADs. The law states that ADs must be respected in all decisions regarding medical treatment irrespective of the stage of the disease and that measures that were clearly specified as unwanted in a patient’s ADs constitute physical assault upon the patient [9, 11]. Since the new law has come into effect, several telephone surveys have been conducted to gather information on the dissemination of ADs in the German population [12–14]. Two of these surveys interviewed individuals of the general adult population aged 18 years and older. Based on samples of *n* = 1044 and *n* = 1005 participants, these surveys showed an ADs completion rate of 26% in 2012 [12] and in 2014 [14]. Both surveys also provided information on the ADs completion rate in different age groups of the population and both reported the highest completion rate for the oldest considered group of individuals aged 60 years and older (42% and 44% respectively [12, 14]). More detailed information on the dissemination of ADs in older and oldest-old age groups was not provided. Completion of ADs, however, becomes naturally much more relevant with older age due to increasing risks of situations requiring serious decisions (e.g., situations of terminal illnesses, end-of-life care) as well as due to increasing risk of reduced or lost decisional capacity (e.g., because of increasing prevalence of cognitive deficits/dementia). Information on completion rates of ADs might be of particular importance for clinical practice and research but also for governments and insurance and health care providers in order to concentrate efforts to extend the dissemination of ADs in targeted population groups.

In this study, we, therefore, aimed at adding to the currently available literature by examining the dissemination of ADs specifically in oldest-old individuals. Based on a large German sample of dementia-free general practitioner (GP) patients aged 85+ years, we sought to provide information on (i) the frequency of ADs for health care, (ii) associated factors, (iii) groups of persons assisting in preparation of ADs, and (iv) reasons for not having ADs in very old individuals. Moreover, as little is known about the frequency of POAs for health care in that oldest-old age group, we sought to provide this information (v) and information on (vi) associated factors, and (vii) the groups of persons empowered as well.

## Methods

### Sampling

We studied general practice patients who participated in the *Study on needs, health service use, costs and health-related quality of life in a large sample of oldest-old primary care patients (85+)* (AgeQualiDe). The AgeQualiDe study is a continuation (follow-up 7 to 9) and extension of the longitudinal *German Study on Ageing, Cognition and Dementia in Primary Care Patients* (AgeCoDe). In this study, we provide cross-sectional results from the baseline wave of AgeQualiDe that is the seventh follow-up of AgeCoDe. During this study wave, information on ADs and POA was assessed. Participants of AgeCoDe were initially recruited via their GP in collaboration with six study centers (Hamburg, Bonn, Düsseldorf, Leipzig, Mannheim and Munich).

GP patients were eligible for AgeCoDe, if they were aged 75 years or older, dementia-free in the GP’s view and had at least one contact with the GP in the prior 12 months. Patients who (i) were not a regular patient of the participating practice, (ii) were consulted only by home visits, (iii) were residents of a nursing home, (iv) had an illness deemed likely to be fatal within 3 months, (v) lacked sufficient facility in German, (vi) were deaf or blind, or (vii) lacked ability to consent were excluded. The study design of AgeCoDe has been described in detail elsewhere [15, 16].

Among a randomly selected sample of 6619 referred GP patients, *n* = 3327 individuals participated in the AgeCoDe study at baseline (50.3%; data collection between January 2003 and November 2004). Among these 3327 individuals, *n* = 868 (26.1%) also participated in the AgeCoDe follow-up wave seven, which corresponds to the AgeQualiDe baseline wave, 11 years later. Most of the remaining individuals had died before this study wave (*n* = 1733; 52.1%); others refused participation/dropped-out or were otherwise unable to participate (not located, moved away, severely ill, etc.; *n* = 726; 21.8%). When compared to AgeCoDe follow-up six in particular, 955 individuals were still alive at AgeCoDe follow-up seven/AgeQualiDe baseline and could have thus been potentially interviewed. The participation of the 868 individuals of these 955 ones at this assessment wave thus corresponds to a response rate of 90.1%.

### Data collection and assessment procedures

Data collection of the AgeQualiDe baseline wave took place between January 2014 and September 2015. All assessments with the participants were performed in participants’ homes by trained physicians or psychologists.

The core assessment instrument was the Structured Interview for Diagnosis of Dementia of Alzheimer type, Multi-infarct Dementia and Dementia of other Aetiology according to DSM-III-R, DSM-IV and ICD-10 (SIDAM) [17]. The SIDAM includes a cognitive test battery, a section for summary clinical diagnostic impression, and a third-party rating of psychosocial impairment with a 14-item scale for the assessment of activities of daily living (SIDAM ADL scale). The cognitive test battery consists of 55 items that cover several domains of cognitive function (orientation, memory, abstract reasoning, verbal abilities and calculation, constructional abilities, aphasia and apraxia). The 55 items include the 30 items of the Mini-Mental State Examination (MMSE) [18].

If SIDAM results were unavailable, we obtained information about a participant’s cognitive status by interviewing an informant using the structured Global Deterioration Scale [19] and “Changes in Performance of Everyday Activities” and “Changes in Habits” subscales from the Blessed Dementia Scale [20].

Subjectively perceived cognitive decline was evaluated prior to cognitive testing. For the purposes of this study, we analyzed data of the question, “Do you feel as if your memory is becoming worse?”(yes/no/I don’t know).

A standardized interview provided information on socio-demographic characteristics such as age, gender, family status, living situation, and education. Education was classified as low, medium, or high according to the revised version of the international new CASMIN educational classification [21].

Difficulties in basic activities of daily living (ADL) were assessed with the 10-item Barthel Index ([22]; total score range = 0–100 points; the higher the score, the better the functioning in basic ADL), difficulties in instrumental ADL with the 8-item Lawton and Brody IADL scale ([23]; total score range = 0–8 points; the higher the score, the better the functioning in instrumental ADL). Frailty was assessed with the Clinical Frailty Scale of the Canadian Study of Health and Aging (CSHA CFS; [24]). CSHA CFS ratings on frailty range from 1 (robust health) to 7 (complete functional dependence on others).

We identified depressive features using the 15-item version of the Geriatric Depression Scale [25] with a cut-off ≥6 [26].

The subjectively perceived current state of health was assessed with the EuroQol visual analogue scale (EQ VAS scale) [27, 28]. The EQ VAS scale ranges from 0 (worst imaginable health state) to 100 (best imaginable health state).

### Advance directives and power of attorney for health care

Information on ADs and POA for health care was assessed via a standardized interview. In the first part, a short description of ADs for health care was provided. Participants were then asked whether they have such ADs (yes/no/don’t know). If participants reported having such ADs, they were additionally asked whether they had received assistance in its preparation (yes/no/don’t know). If participants reported having had help, they were asked who had provided the help (a predefined list of possible groups of persons who could have assisted was provided along with the opportunity to state further persons). If participants reported not having ADs, they were additionally asked about possible reasons (a predefined list of possible reasons for not having ADs for health care was provided along with the opportunity to state other reasons). In the second part, a short description of a POA for health care was provided. As with the ADs part, participants were then asked whether they had such a POA (yes/no/don’t know). If participants reported having a POA, they were additionally asked who had been empowered (a predefined list of possible groups of persons who could have been empowered was provided along with the opportunity to state further persons).

Dates of death were obtained from relatives, GPs, or from the local registry offices where all persons in Germany must be registered in accordance with German law.

### Diagnosis of dementia

All diagnoses of dementia were based on DSM-IV criteria [29] and made in consensus conferences that included the trained interviewer (physicians or psychologists) of the participant and/or the informant and an experienced geriatric psychiatrist or geriatrician who had not met the participant/informant but supported the interviewer in reviewing all collected information to judge whether a dementia diagnosis should or should not be assigned. If a participant could be interviewed personally, dementia diagnosis was assigned mainly based on information derived from the SIDAM [17] (see above) that incorporates a standardized diagnostic algorithm of dementia according to the DSM-IV criteria [29]. If a participant could not be interviewed personally and SIDAM results were unavailable (e.g. in case of death), then, as mentioned above, standardized interviews with informants (usually relatives) were conducted including the structured Global Deterioration Scale [19] and “Changes in Performance of Everyday Activities” and “Changes in Habits” subscales from the Blessed Dementia Scale [20]. In such cases, dementia diagnosis was assigned in the consensus conference mainly based on results in these subscales (ratings ≥4 on the Global Deterioration Scale [19] and/or >8 on the Blessed Dementia Rating subscales [20]).

### Statistical analysis

The statistical analyses were performed using the Statistical Package for the Social Sciences (SPSS; version 20.0). All analyses employed an alpha level for statistical significance of 0.05 (two-tailed). Group differences were analyzed using t-test, Mann-Whitney-U-test, and χ^2^-test as appropriate.

The frequency of having ADs and a POA for health care in the sample of oldest-old primary care patients was stated in percentages with 95% confidence intervals (95%-CI). To evaluate the association between having ADs and a POA for health care and participants’ characteristics, we then used multivariable logistic regression analyses (backward elimination). We included the variables age, gender, education, family status, living situation, subjectively perceived cognitive decline, MMSE scores, self-rated health, depression, frailty, and basic and instrumental functional status in the regression models because we believed they could have a potential relationship to having ADs and POA. For each of the variables, we calculated odds ratios (ORs) and 95%-CI.

## Results

### Characteristics of the sample

Among the 868 GP patients who participated in the baseline wave of AgeQualiDe, *n* = 86 did not attend the interview section on ADs and POA for health care as they had either dementia (*n* = 83) or were too exhausted or too ill to answer the questions (*n* = 3). Another *n* = 78 patients did answer the interview questions on ADs and POA, but were also classified as having dementia. We excluded these participants from analyses as we cannot be sure that they completely understood the content of the questions, leaving a final analysis pool of *n* = 704 dementia-free elderly individuals, 472 (67.0%) women and 232 (33.0%) men. The mean age of the sample was 88.7 years (SD = 3.0).

### Advance directives for health care in oldest-old age

#### Frequency of advance directives for health care in oldest-old age and associated factors

Among the *n* = 704 participants who answered questions on ADs and POA for health care, *n* = 486 (69.0%; 95%-CI = 65.6–72.4) reported having ADs and *n* = 192 (27.3%; 95%-CI = 24.0–30.6) to not having ADs. The remaining *n* = 26 (3.7%; 95%-CI = 2.3–5.1) participants did not know whether they had or had not completed ADs. As shown in Table 1, participants with and without ADs did not differ significantly in age, gender, family status, living situation, subjectively perceived cognitive decline, self-rated health, depression, frailty, and basic and instrumental functional status. Significant differences, however, were found in education and MMSE scores. Conducting a multivariable logistic regression analysis (backward elimination) on the association between having ADs for health care and the participants’ characteristics, these two variables were accordingly left in the model (see Table 2): Every additional point on the MMSE yielded an OR =1.15 (95%-CI = 1.05–1.26) and medium versus low education an OR =1.56 (95%-CI = 1.05–2.30) for having ADs for health care.

**Table 1:** Characteristics of the study participants with and without ADs and POA for health care

Characteristic	Study participants with and without ADs (*n* = 678)				Study participants with and without POA (*n* = 666)			
	Individuals with ADs (*n* = 486)	Individuals without ADs (*n* = 192)	Test values	*p*	Individuals with POA (*n* = 455)	Individuals without POA (*n* = 211)	Test values	*p*
Age								
Years, mean (SD)	88.6 (2.9)	88.8 (3.1)	*t* = 0.652	0.676	88.6 (2.9)	88.8 (3.0)	*t* = 0.717	0.474
Gender; n (%)								
Female	327 (67.3)	126 (65.6)	χ^2^ = 0.171	0.679	308 (67.7)	137 (64.9)	χ^2^ = 0.496	0.481
Male	159 (32.7)	66 (43.4)			147 (32.3)	74 (65.1)		
Education^a^; n (%)								
Low	251 (51.6)	121 (63.0)	χ^2^ = 7.194	0.027	239 (52.5)	125 (59.2)	χ^2^ = 2.686	0.261
Medium	163 (33.5)	49 (25.5)			149 (32.7)	58 (27.5)		
High	72 (14.8)	22 (11.5)			67 (14.7)	28 (13.3)		
Family status; n (%)								
Single	33 (6.8)	12 (6.2)	χ^2^ = 2.893	0.408	27 (5.9)	17 (8.1)	χ^2^ = 1.814	0.612
Married	123 (25.3)	46 (24.0)			116 (25.5)	49 (23.2)		
Divorced	26 (5.3)	5 (2.6)			20 (4.4)	12 (5.7)		
Widowed	304 (62.6)	129 (67.2)			292 (64.2)	133 (63.0)		
Living situation; n (%)								
Private household, alone	246 (50.6)	110 (57.3)	χ^2^ = 3.692	0.158	224 (49.2)	124 (58.8)	χ^2^ = 8.032	0.018
Private household, with others (partner, relative, etc.)	153 (31.5)	58 (30.2)			146 (32.1)	64 (30.3)		
Assisted living/nursing home/retirement home	87 (17.9)	24 (12.5)			85 (18.7)	23 (10.9)		
Subjective Cognitive Decline; n (%)								
Yes	301 (61.9)	118 (61.5)	χ^2^ = 0.047	0.977	276 (60.7)	131 (62.1)	χ^2^ = 0.132	0.936
MMSE^b,c^								
Score, mean (SD)	28.1 (1.8)	27.6 (1.9)	*t* = −3.045	0.002	28.1 (1.7)	27.6 (1.9)	*t* = −3.506	0.001
Self-rated health^d,e^								
EQ VAS score, mean (SD)	62.1 (18.9)	63.3 (18.6)	*t* = 0.737	0.461	62.5 (19.0)	62.0 (18.3)	*t* = −0.286	0.775
Depression^f,g^; n (%)								
Yes	73 (15.1)	24 (12.5)	χ^2^ = 0.746	0.388	63 (13.9)	31 (14.7)	χ^2^ = 0.037	0.787
Frailty^h^								
CSHA CFS score; mean (SD)	3.8 (1.4)	3.8 (1.4)	U = 46,378.500	0.902	3.8 (1.4)	3.8 (1.3)	U = 47,419.000	0.796
Basic ADL^i,j^								
Barthel Index score; mean (SD)	92.6 (12.9)	93.2 (12.2)	U = 45,808.500	0.714	92.8 (13.1)	92.2 (12.7)	U = 45,469.500	0.286
Instrumental ADL^k^								
IADL score; mean (SD)	6.2 (1.9)	6.3 (1.9)	U = 44,498.500	0.333	6.2 (1.9)	6.3 (1.9)	U = 46,996.000	0.653

*AD* advance directives, *ADL* activitities of daily living, *CSHA CFS* Canadian Study of Health and Aging Clinical Frailty Scale, *EQ VAS* EQ visual analogue scale, *IADL* instrumental activities of daily living, *MMSE* Mini-Mental-State Examination, *POA* power of attorney, *U* Mann-Whitney U

^a^according to the new CASMIN educational classification [21]

^b^missing data for *n* = 14 (2.1%) participants in the analysis for ADs and *n* = 13 (2.0%) in the analysis for POA

^c^The higher the MMSE score, the better the cognition [18]

^d^missing data for *n* = 8 (1.2%) participants in the analysis for ADs and *n* = 8 (1.2%) in the analysis for POA

^e^The higher the EQ VAS score, the better the self-rated health [27, 28]

^f^missing data for *n* = 2 (0.3%) participants in the analysis for ADs *n* = 2 (0.3%) in the analysis for POA

^g^a score ≥ 6 on the Geriatric Depression Scale [25, 26]

^h^The higher the CSHA CFS score, the stronger the frailty [24]

^i^missing data for *n* = 1 (0.1%) participants in the analysis for ADs and *n* = 2 (0.3%) in the analysis for POA

^j^The higher the score, the better the functioning in basic ADL [22]

^k^The higher the score, the better the functioning in IADL [23]

**Table 2:** Multivariable logistic regression on the association between having ADs for health care and participants’ characteristics^a, b^

Characteristic^c^	df	Wald’s χ^2^	*p* value	OR	95%-CI
Education^d^					
Medium vs. low	1	4.886	0.027	1.56	1.05–2.30
High vs. low	1	1.999	0.157	1.47	0.86–2.50
MMSE score; every additional point^e^	1	8.955	0.003	1.15	1.05–1.26

*CI* confidence interval, *df* degree of freedom, *MMSE* Mini-Mental-State Examination, *OR* odds ratio

^a^missing data for *n* = 23 (3.9%) of the 678 participants

^b^backward elimination; Nagelkerke’s R^2^ of the model =0.034

^c^Age, gender, family status, living situation, subjectively perceived cognitive decline, self-rated health, depression, frailty, and basic and instrumental functional status were excluded from the model

^d^according to the new CASMIN educational classification [21]

^e^The higher the MMSE score, the better the cognition [18]

#### Reasons for not having advance directives for health care

As shown in Table 3, the most frequently stated reasons for not having ADs for health care were that the participants trust their relatives (59.4%) or their physicians (44.8%) in making the right decisions for them when necessary. The third most frequently stated reason was that the participants just did not want to concern themselves with the topic of ADs (28.1%). Participants also frequently stated that they did not have the right contact person or help to prepare ADs (23.4%), and that the topic of ADs is too complicated (19.3%). Also, 12.5% of the participants stated that they were not aware of the possibility of preparing ADs.

**Table 3:** Reasons for not having advance directives for health care (*n* = 192)

Reason	Stated by n (%) of the 192 participants without ADs^a^
“I trust that my relatives will make the right decisions for me.”	114 (59.4)
“I trust that my physicians will make the right decisions for me.”	86 (44.8)
“I did not want to concern myself with the topic ADs.”	54 (28.1)
“I did not have the right contact person or help to prepare an ADs.”	45 (23.4)
“The topic ADs is too complicated for me.”	37 (19.3)
“I have not had the time to deal with the topic ADs so far.”	32 (16.7)
“I have too many concerns regarding the usefulness of an ADs.”	32 (16.7)
“I was not aware of the possibility of preparing an ADs.”	24 (12.5)
Other reasons^b^	60 (31.3)
No reasons stated	4 (2.1)

*ADs* advance directives

^a^Multiple answers were allowed

^b^common stated other reasons: preparation of ADs is planned/intended (*n* = 13; 6.8%); no motivation/laziness/carelessness/indifference (*n* = 19; 9.9%), ADs are not necessary (*n* = 12; 6.3%)

#### Assistance in preparation of advance directives

Regarding those 486 participants who stated that they have ADs for health care, *n* = 95 (19.5%) declared that they prepared the directives on their own and *n* = 384 (79.0%) stated that they received assistance in its preparation (missing data for the remaining *n* = 7/1.4% participants). As shown in Table 4, most participants stated that they received assistance from their children or grandchildren (38.3%), followed by notaries (24.2%), and general practitioners (19.0%).

**Table 4:** Overview of the groups of persons who assisted in the preparation of advance directives

Groups of persons assisting in ADs preparation	Assisted in n (%) of the 384 participants with assistance in ADs preparation^a^
Children/grandchildren	147 (38.3)
Notary	93 (24.2)
General practitioner	73 (19.0)
Spouse/life partner	51 (13.3)
Other relatives	40 (10.4)
Acquaintance/friend	24 (6.3)
Staff of professional information centers^b^	20 (5.2)
Lawyer	6 (1.6)
Others^c^	6 (1.6)

*ADs* advance directives

^a^Multiple answers were allowed

^b^e.g., health insurances, social welfare organizations

^c^e.g., tax accountant, staff of nursing or retirement home, pastor

### Power of attorney for health care in oldest-old age: Frequency, associated factors, empowered persons

Among the *n* = 704 participants with completed information on ADs and POA for health care, *n* = 455 (64.6%; 95%-CI = 61.1–68.2) reported having a POA and *n* = 211 (30.0%; 95%-CI = 26.6–33.4) reported not to having a POA. The remaining *n* = 38 (5.4%; 95%-CI = 3.7–7.1) participants did not know whether or not they had completed a POA. As shown in Table 1, participants with and without a POA did not differ significantly in age, gender, education, family status, subjectively perceived cognitive decline, self-rated health, depression, frailty, and basic and instrumental functional status. Significant differences, however, were found in living situation and MMSE scores. Conducting a multivariable logistic regression analysis (backward elimination) on the association between having a POA for health care and the participants’ characteristics, these two variables were accordingly left in the model (see Table 5): Every additional point on the MMSE yielded an OR =1.21 (95%-CI = 1.10–1.33) and assisted living or living in nursing or retirement home versus living alone in a private household an OR = 2.15 (95%-CI = 1.26–3.66) for having POA.

**Table 5:** Multivariable logistic regression on the association between having a POA for health care and participants’ characteristics^a, b^

Characteristic^c^	df	Wald’s χ^2^	*p* value	OR	95%-CI
Living situation (reference: private household, alone)					
Private household, with others (partner, relative, etc.)	1	1.844	0.175	1.30	0.89–1.89
Assisted living/nursing home/retirement home	1	7.959	0.005	2.15	1.26–3.66
MMSE score; every additional point^d^	1	16.054	<0.001	1.21	1.10–1.33

*CI* confidence interval, *df* degree of freedom, *MMSE* Mini-Mental-State Examination, *OR* odds ratio

^a^missing data for *n* = 22 (3.3%) of the 666 participants

^b^backward elimination; Nagelkerke’s R^2^ of the model =0.051

^c^Age, gender, education, family status, subjectively perceived cognitive decline, self-rated health, depression, frailty, and basic and instrumental functional status were excluded from the model

^d^The higher the MMSE score, the better the cognition [18]

Regarding those 455 individuals who responded as having a POA for health care, most declared that they empowered their children or grandchildren (*n* = 349; 76.7%; multiple answers were allowed). The spouse or life partner was stated as attorney by *n* = 49 (10.8%), another relative by *n* = 63 (13.8%), and acquaintances or friends by *n* = 25 (5.5%) individuals. Other persons were stated as attorney in *n* = 8 cases (1.8%; e.g., lawyer, general practitioner, staff of retirement home) cases. *N* = 5 (1.1%) individuals could not state, which person they had empowered.

### Overlap of having of advance directives and power of attorney for health care in oldest-old age

In a last step, we also analyzed the overlap of having ADs and a POA for health care in oldest-old age. Among the *n* = 704 participants with available information on ADs and POA, *n* = 411 (58.4%; 95%-CI = 54.7–62.0) stated having both ADs and POA for health care, *n* = 58 (8.2%; 95%-CI = 6.2–10.3) to have ADs but not POA, and *n* = 40 (5.7%; 95%-CI = 4.0–7.4) to have POA but not ADs. Another *n* = 17 (2.4%; 95%-CI = 1.3–3.5) participants stated to having ADs but did not know whether or not they had completed a POA. Vice versa, *n* = 4 (0.6%; 95%-CI = 0.1–1.1) participants stated that they had a POA for health care but did not know whether they had completed ADs or not. Overall, *n* = 530 (75.3%; 95%-CI = 72.1–78.5) participants stated to have at least one either ADS or POA, whereas *n* = 148 (21.0%; 95%-CI = 18.0–24.0) claimed to have neither ADs nor a POA for health care. For the remaining *n* = 26 (3.7%; 95%-CI = 2.3–5.1) participants, we do not know whether they had at least one of the two tools.

### Excursus: Advance directives and power of attorney for health care in oldest-old individuals with dementia

In a subsample of *n* = 102 AgeCoDe/AgeQualiDe participants who had dementia at the AgeQualiDe baseline wave or who had dementia but died before the baseline wave, we were also able to collect information on the presence/absence of ADs and POA by conducting additional proxy interviews. The observed ADs completion rate was 67.6% (95%-CI = 58.6–76.7; *n* = 69) and the observed POA completion rate was 71.6% (95%-CI = 62.8–80.3; *n* = 73). A proportion of 77.5% (95%-CI = 69.3–85.6; *n* = 79) had at least one of the two tools, whereas 16.7% (95%-CI = 9.4–23.9; *n* = 17) had neither ADs nor POA for health care (*n* = 6/5.9% missing information).

## Discussion

In this study, we specifically aimed to provide information on the dissemination of *advance directives* (ADs) and *power of attorney* (POA) for health care in oldest-old individuals. Based on a German sample of *n* = 704 dementia-free general practitioners’ (GP) patients aged 85+ years, we found that 69.0% stated to having ADs and 64.6% to having a POA for health care. As more than 90% of elderly people in Germany have regular contact with a GP [30], our findings may be a good indicator of the dissemination of these tools in the oldest-old population in Germany in general.

So far, available information on the dissemination of the two health care planning tools in older age groups of the population in Germany was mainly limited to (i) ADs (and not POA) in (ii) individuals aged 60+ or 65+ years (and not specifically in older individuals). As stated earlier, for instance, two more recent telephone surveys (conducted in 2012 and 2014 [12, 14]) reported AD completion rates of 42% and 44% for the age group 60 years and older (as well as completion rates of 26% for the German general adult population aged 18 years and older). Importantly, when compared with findings from older surveys, these and other findings (e.g. [9, 31]) strongly indicate an increase in ADs completion rates in Germany over the last years. Regarding two representative surveys that were conducted in 2006/2007 with samples of *n* = 400 and *n* = 1000 individuals, for example, ADs completion rates of 18.0% and 18.1% for individuals aged 65+ years (and of 11.0% and 10.4% for individuals aged 16–92 years) were found [32]. Even though we did not find other studies providing information on ADs completion rates in very old individuals in Germany (as was the focus of our study), we suggest that our observed completion rate of nearly 70% in the oldest-old dementia-free GP patients may additionally support the notion that the dissemination of ADs in the German older adult population is generally increasing.

Upon reviewing the international literature, most information on completion rates of ADs can be derived from studies from the U.S., likely because research in this field was strongly stimulated by the *Patient Self-Determination Act*, a law that came into effect in 1991. This legislation (amongst others) requires most health care facilities in the U.S. to inform adult patients about their right to complete ADs [33]. Regarding the U.S. general adult population, a current study from Rao et al. [34] analyzed data from *n* = 7946 participants (aged 18+ years) from a national mail survey conducted in 2009/2010. Results showed an ADs completion rate of 26.3%, a rate that is similar to the rate reported for the German general adult population by the above mentioned telephone surveys [12, 14]) but higher than, for example, the rate that was currently observed for the Australian general adult population (14.4%; data derived from a national telephone survey conducted in 2012; *n* = 2405 [5]). Regarding the older population in particular, Rao et al. [34] reported an ADs completion rate of 51.2% for American adults aged 65 years and older. Moreover, findings from a current review on literature published between 2008 and 2013 on ADs among U. S. older adults indicated an even slightly higher rate of 55–60% in this age group [35]. And finally, a recent study from the U.S. that analyzed data from a retrospective cohort study (*n* = 6122) indicated a significant increase in ADs and POA completion rates between 2000 and 2010. The proportion of individuals who had ADs, POA or both increased from 47% to 72% at the end of life for elderly Americans (participants were aged 60 years and older at death). However, the study identified lesser effects of this increase with regard to rates of hospitalization and hospital death [4].

In our study, we found that three-out-of-four dementia-free older GP patients (75.3%) reported having completed least one of the two tools – a rate that is similar to that reported by Silveira et al. [4] for elderly Americans, although our study surveyed adults of a significantly higher age (mean/SD = 88.7/3.0 years vs. 81.0/9.6 vs. years). It can be argued that the high ADs/POA completion rates in a younger age group in the study by Silveira et al. [4] might be due to the fact that their findings are based on data (from proxy interviews) for decedents, and thus, on data for individuals who might have had already health care situations, in which ADs/POA were prudent. The decedents, however, on average, completed the ADs and POA documents a relatively long time before their death (ADs: mean = 54.6 months: POA = 40.3 months), therefore impending death might not be the only reason for the high completion rate. It is also likely that a high awareness of the issue ADs/POA in the American older adult population (also stimulated by the *Patient Self-Determination Act* from 1991) might have contributed to the high rate, as Silveira et al. [4] have shown a significant increase in ADs/POA completion from 2000 (47%) to 2010 72%).

Regarding our study findings, lack of awareness seems not to be the main reason for not having the two considered tools in oldest-old age in Germany. Specifically, only 12.5% of those participants without ADs stated that they were not aware of the possibility of preparing such directives. Individual characteristics of the oldest-old GP patients were also of limited use in predicting the presence/absence of ADs and POA for health care in our study. Having completed ADs was only associated with medium education (reference low) and higher global cognitive functioning in terms of a higher MMSE score and having a POA only with assisted living/living in a nursing or retirement home (reference alone in private household) and with higher global cognitive functioning as well. However, the Nagelkerke’s R^2^ values of the final multivariable regression models were low (0.034 and 0.051) implying that individual characteristics explained only a very small variance of whether or not an individual had completed ADs or a POA.

Irrespective of the low R^2^ values, our findings corroborate findings of others [36, 37] that older adults with cognitive impairment, who have a substantially increased risk for (future) decisional incapacity, lack relevant health care planning and may be of particular need for tailored interventions to enhance the completion of advance care documents. These findings may be also supported by our observed ADs and POA completion rates in a subsample of oldest-old individuals with dementia (e.g., 16.7% had neither ADs nor POA for health care; see Excursus above) – a sample of individuals, who for the vast proportion had already lost their own decisional capacity for health care situations.

Regarding the limited use of individual characteristics in predicting the presence/absence of ADs and POA for health care, it is possible that other characteristics that could not be considered in our study might have had a more significant impact. Previous studies, for example, identified completion of ADs to be also positively associated with characteristics like higher income [34], having a regular source of care [34], preparation of other planning documents [5], knowing someone with cognitive impairment [38], and having not recently experienced the death of a relative or acquaintance [32, 35]. The impact of such characteristics, however, was investigated only in few or single studies. Characteristics that were investigated more frequently and more consistently found to be associated with ADs completion are rather those that lacked association in our study including older age (e.g. [9, 34, 35, 38]), female gender (e.g. [21, 35, 38]), not living in the community (e.g. [35]), and declining health/experience a serious/chronic disease (e.g. [9, 34, 35]; comparable to our included characteristics of subjectively perceived cognitive decline, self-rated health, depression, frailty, and basic and instrumental functional status). Another important group of characteristics that could not be considered in our study but likely also significantly contributes to presence/absence of ADs and POA for health care in oldest-old age may be spiritual and cultural beliefs about death and dying. A recent review by Fang et al. [39], for example, identified numerous such beliefs impacting end-of-life decision making including, for instance, the degree of belief that God may control the timing and nature of death, or identified social and cultural taboos (e.g., the idea that speaking about death may bring upon death). Further studies are strongly recommended to analyze the impact of such beliefs on completion/non-completion of ADs/POA documents in oldest-old age.

It is, on the other hand, therefore also possible that having/not having ADs and POA in oldest-old age may be generally less dependent on individual (sociodemographic and health-related) characteristics and more dependent on attitudinal factors. Regarding our findings, the most frequently stated reason for not having ADs for health care, for example, was that the respondents trust their relatives or physicians to make the right decisions for them when necessary (stated by 59.4% and 44.8% of those without ADs). The high importance of trust, particularly in relatives, corroborates findings of Rao et al. [34] which found that for the older U.S. population, trust in relatives (“my family knows my wishes”) was the second most frequently (16.4%) stated reason for not having ADs after lack of awareness of the possibility of preparing such ADs (24%). As mentioned above, lack of awareness was not found to be a main reason for not having ADs in our study (stated only by 12.5% of those without ADs). In line with previous findings [35], identified important attitudinal reasons, for example, were that oldest-old respondents do not want to concern themselves with the topic ADs (28.1%), or have too many concerns regarding the usefulness of ADs (16.7%). Moreover, procrastination seems to play also an important role in not having ADs in our oldest-old age sample. We found that 16.7% of respondents without ADs stated that they did not have the time to deal with the topic of ADs so far and 6.8% stated that preparation of ADs is planned/intended.

An individual’s wishes should be respected as part of protecting an individual’s autonomy. If individuals clearly do not want to concern themselves with the topic ADs and trust their relatives/physicians to make the right decisions for them when necessary, or if individuals clearly do not want to complete ADs and other advance care documents because of too many concerns about the usefulness of such documents, these wishes must also be respected. For other oldest-old individuals who may be open to completing such documents, but stated reasons for not having ADs such as, *“I did not have the right contact person or help to prepare ADs.“* (23.4%) or *“The topic ADs is too complicated for me.”* (19.3%), however, support should be offered. As more than 90% of elderly people in Germany have regular contact with a GP [30], GPs may be the first contact person for such support within the German health care system. Current findings suggest that the implementation of a systematic *advance care planning* (ACP) program (“beizeiten begleiten®”; based on the US ACP program “Respecting Choices®” [40]) in German nursing homes led to an increasing completion of ADs documents with potential relevance to medical decision-making [7]. The program included: educative sessions for nursing staff, medical and paramedic emergency staff, and professional guardians; training of non-physician facilitators from participating nursing homes; and training for cooperating GPs. To the best of our knowledge, controlled trials are pending to investigate whether systematic ACP can be implemented to effectively increase completion of advance care documents also in the community-dwelling older population of Germany. Such trials are strongly encouraged not only by the promising findings on ACP implementation in German nursing homes [7], but also by promising findings derived from an US study. Results from this study indicate that the implementation and continuous improvement of a systematic ACP approach (“Respecting Choices®” [40]) within a geographic region (La Crosse County, Wisconsin, USA) can achieve a high prevalence of ADs documents (90%) and a high availability of these documents in the medical records (99.4%) at the time of death [6]. Findings, moreover, indicated a high clarity and specificity of the advance care documents, as well as high consistency between the advance care plans and medical treatments.

Our study is not without limitations. First, information on the presence or absence of ADs and POA for health care was mainly self-reported by the oldest-old individuals. We attempted to carefully assess information on ADs and POA in this very old age group by conducting structured interviews and providing short and easy to understand descriptions of the two tools. Further, we excluded from analysis data of participants who we were not sure whether they understood or not the content of the questions. However, it cannot be completely guaranteed that all participants understood, for example, the difference between ADs and POA for health care. Moreover, due to a generally increasing awareness of ADs and POA, having one or both of these tools might be considered socially more desirable than not having one. It cannot be excluded that some participants without ADs and/or POA responded in this socially more desirable manner. Not less important, clinical experience suggests that a proportion of older adults who state that they have ADs and/or POA are not able to retrieve the document(s) in their files. Thus, our reported ADs and POA completion rates might be rather over- than underestimates of the real rates. Second, regarding the analysis of the associations between participant characteristics and ADs and POA completion rates, we were only able to provide cross-sectional results. The associations, however, might be time-sensitive and it is therefore possible that an analysis of longitudinal data collected in a period lasting several years might have revealed an impact of a certain factor that was not found in cross-sectional analysis and vice versa. Moreover, as addressed above, there is large body of characteristics that may potentially contribute to completion/non-completion of ADs/POA documents in oldest-old age. We aimed to investigate the impact of several such important characteristics including socio-demographic, cognitive, functional and health-related aspects, but our assortment was limited to those assessed in the AgeCoDe/AgeQualiDe study and therefore not included, for example, were spiritual and cultural beliefs of death and dying that may have also contributed. Third, we were not able to assess reasons for not having POA for health care. It can be assumed that the reasons did not differ substantially from the identified reasons for not having ADs, but this is just an assumption. Finally, and importantly, we cannot provide information on the content (i.e. meaningfulness) or validity of the ADs/POA documents of the participants (i.e., for example, on whether ADs relate to clinically relevant choices or on whether stated preferences truly reflect advance informed consent).

## Conclusions

Irrespective of these limitations, we believe that by providing information specifically on the dissemination of advance directives (ADs) and power of attorney (POA) for health care in oldest-old individuals, our study adds significantly to the currently available literature. Our findings suggest a high dissemination of these tools in oldest-old age in Germany. On the other hand, our findings suggest that the majority of individuals who do not have advance care documents in oldest-old age simply trust their relatives/physicians in making the right decisions for them when necessary, do not want to concern themselves with the topic, or just have too many concerns regarding the usefulness of such an documents. However, we also found a relevant proportion of older adults who actively articulated that they would need more explanation and support for drawing up an advance care document. When planning programs to offer ACP to the oldest old, it might be helpful to relate to these specific needs, as well as to be responsive to attitudinal differences of this target group.

## Abbreviations

ACP: Advance care planning
ADs: Advance directives
ADL: Activities of daily living
AgeCode: German Study on Ageing, Cognition and Dementia in Primary Care Patients
AgeQualiDe: Study on needs, health service use, costs and health-related quality of life in a large sample of oldest-old primary care patients
CI: Confidence interval
CSHA CFS: Clinical Frailty Scale of the Canadian Study of Health and Aging
EQ VAS scale: EuroQol visual analogue scale
GP: General practitioner
IADL: Instrumental activities of daily living
MMSE: Mini-Mental State Examination
OR: Odds ratio
POA: Power of attorney
SIDAM: Structured Interview for Diagnosis of Dementia of Alzheimer type, Multi-infarct Dementia and Dementia of other Aetiology according to DSM-III-R, DSM-IV and ICD-10
